# Chemical and structural analysis on magnetic tunnel junctions using a decelerated scanning electron beam

**DOI:** 10.1038/s41598-018-25638-8

**Published:** 2018-05-15

**Authors:** Edward Jackson, Mingling Sun, Takahide Kubota, Koki Takanashi, Atsufumi Hirohata

**Affiliations:** 1 Department of Electronic Engineering, University of York, Heslington, York, YO10 5DD United Kingdom; 2 Institute for Materials Research, Tohoku University, 2-1-1 Katahira, Sendai, 980-8577 Japan; 3 Center for Spintronics Research Network, Tohoku University, 2-1-1 Katahira, Sendai, 980-8577 Japan

## Abstract

Current information technology relies on the advancement of nanofabrication techniques. For instance, the latest computer memories and hard disk drive read heads are designed with a 12 nm node and 20 nm wide architectures, respectively. With matured nanofabrication processes, a yield of such nanoelectronic devices is typically up to about 90%. To date the yield has been compensated with redundant hardware and software error corrections. In the latest memories, approximately 5% redundancy and parity bits for error corrections are used, which increase the total production cost of the devices. This means the yield directly affects the device costs. It is hence critical to increase the yield in nanofabrication. In this paper, we have applied our recently developed method to image buried interfaces in combination with chemical analysis to evaluate magnetic tunnel junctions and have revealed their different magnetoresistance ratios caused by the presence of materials formed at the junction edges. The formation of these materials can be avoided by optimising the junction patterning process to remove residual carbon introduced from resist. Our imaging method with chemical analysis have demonstrated a significant potential for the improvement of junction performance, resulting in higher yields. This can be used as a quality assurance tool in a nanoelectronic device production line.

## Introduction

Human beings have been reported to have created 4.4 ZB data in 2013 and are expected to create 44 ZB in 2020^1^. Such drastic increase of 40% annually in the digital universe is based on the development of nanoelectronic devices, *e.g*., data processing chips with a 12 nm node and hard disk drives with a 20 nm wide read heads. The latest development on 3-dimensional (3D) NAND flash memories have also achieved 512 Gbit on a single Si die with the area of around 130 mm^2^ independently demonstrated by Toshiba-Western Digital and Samsung^2^. However, the miniaturisation of the nanoelectronic devices have in turn reduced their yields due to defects and non-uniformity induced during their complicated fabrication processes. The yield ($Y$) can be defined as

1 $$Y=\frac{({\rm{Number}}\,{\rm{of}}\,{\rm{working}}\,{\rm{devices}}\,{\rm{on}}\,{\rm{a}}\,{\rm{die}})}{({\rm{Number}}\,{\rm{of}}\,{\rm{total}}\,{\rm{devices}}\,{\rm{fabricated}}\,{\rm{on}}\,{\rm{a}}\,{\rm{die}})}$$

It is widely known that the final device yield can be approximately 90% for a series of optimised processes in nanoelectronics. The exponential yield model gives the following relationship:

2 $$Y=\exp (-\sqrt{AD}),$$

where $A$ and $D$ represent area and defect density of a device fabricated, respectively. In Eq. (2), if $D$ is assumed to be the same on a die, the reduction in $A$ increases $Y$. In reality, the milling or etching processes to fabricate a nanometre-scale junctions induce additional edge defects, such as redeposition of materials and edge roughness distributions, which increases $D$.

In order to compensate a 90% yield to reduce the cost of the device production, hardware redundancy^3^ has been implemented in a device. For example, one of the most common redundancy in a memory is a built-in self-repair technique, which consumes about 5% of the memory. It runs a test of the memory functionality and then analyse redundancy, followed by the allocation of redundant memories for the replacement of a broken section on the memory. In some devices, laser-assisted repair function is implemented to physically repair the broken section. Software error corrections^4^ are also widely utilised in a device input/output interface with additional parity bits. The hardware solution is one-time repair requiring additional technology but it can repair the device in a short time. On the other hand, the software solution is multiple-time repair with almost no design overhead but it can repair only limited latent defects requiring additional processing data time. Therefore, it is crucial to develop a revolutionary method to optimise the device structures to achieve a yield of almost 100%.

Further improvement of the yield requires intensive process optimisation fed back from techniques such as cross-sectional transmission electron microscopy (TEM) imaging etc. TEM observation allows for atomic scale studies into device structure, but the images may not be representative of the device interfaces because of the destructive sample preparation inducing additional strain and defects to the interfaces. Other options are to investigate devices with photoemission electron microscopy (PEEM). This relies on EM radiation to excite or liberate electrons which then provide information about the structure. Unfortunately this is very surface sensitive so is not suited for these kind of studies without intensive sample preparation similar to TEM. In this study, we have demonstrated chemical analysis alongside decelerated electron-beam imaging for buried magnetic tunnel junctions (MTJs) to reveal the origin of the reduction in tunnelling magnetoresistance (TMR) ratios for some of the MTJs. We have recently developed a new non-destructive method to image buried junctions using a decelerated electron-beam^5^. By using a precisely controlled beam we have managed to image MTJs below an 80-nm-thick Au electrode, allowing a correlation between the junction images and their magnetic transport properties to be made. The reduction in their TMR ratios have been found to be induced by the formation of aluminium carbide in resist during Ar-ion milling to pattern MTJ pillars. This has been fed back to the fabrication process for the optimisation and can be applicable for the other nanoelectronic devices.

## Results

### Magnetic transport properties

**Figure 1:**
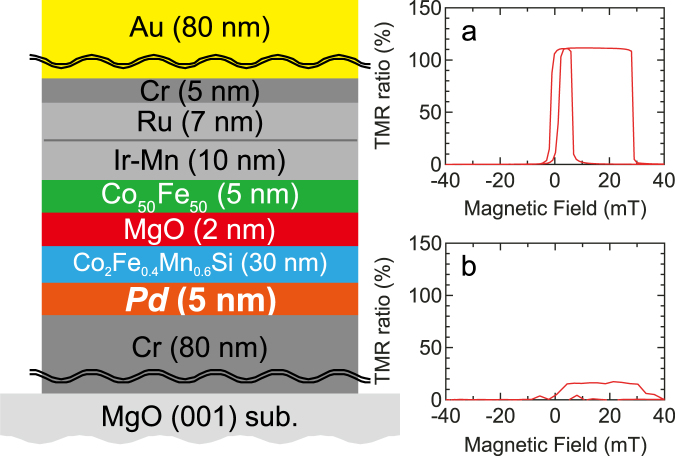
Magnetic tunnel junctions studied. Multilayered structure of MgO(001)//Cr (80)/Pd (5)/Co_2_Fe_0.4_Mn_0.6_Si (30)/MgO (2)/Co_0.5_Fe_0.5_ (5)/IrMn_3_ (10)/Ru (7)/Cr (5)/Au (80) (thickness in nm). The multilayers were patterned into magnetic tunnel junctions (MTJs) by photolithography and Ar-ion milling. MTJs were measured by a four-terminal method, showing a typical tunnelling magnetoresistance (TMR) ratio and resistance-area product (*RA*) of (**a**), 110% and 3.8 × 10^4^ Ω µm^2^ and (**b**), 18% and 4.7 × 10^3^ Ω µm^2^, respectively. In the supplementary section there are also results shown from a similar set of devices where CFMS = 5 nm.

Many MTJs, consisting of MgO(001)//Cr (80)/Pd (5)/Co_2_Fe_0.4_Mn_0.6_Si (30)/MgO (2)/Co_0.5_Fe_0.5_ (5)/IrMn_3_ (10)/Ru (7)/Cr (5)/Au (80) (thickness in nm), were measured by a four-terminal method. The results of these measurements are shown in Supplementary Table ST1. Two distinctive groups of MTJs were found in terms of their TMR ratios: (i) A large TMR ratio of over 80% and (ii) a small TMR ratio of about 20% as shown in Fig. 1. Their resistance-area product (*RA*) were also found to be as different as 4.2 × 10^4^ Ω µm^2^ for the group (i) and 4.7 × 10^3^ Ω µm^2^ for the group (ii). The average difference between these groups was approximately one order of magnitude.

### Electron flight simulations

**Figure 2:**
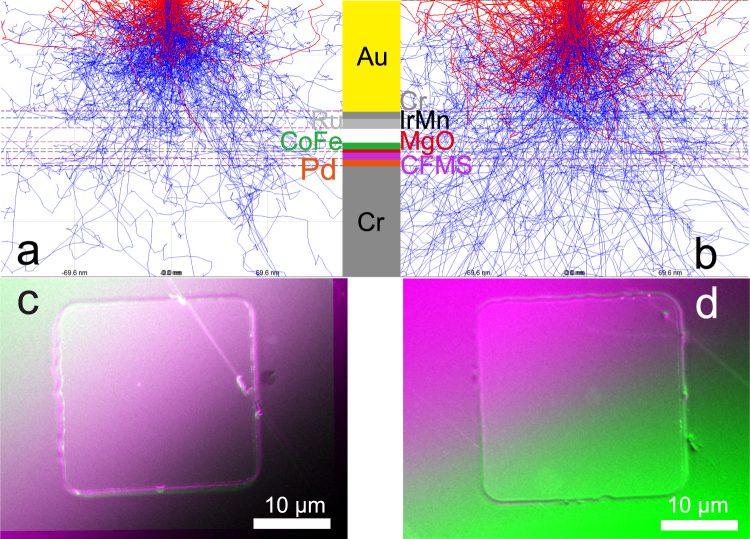
Simulations and SEM images of MTJs with decelerated electron beam. Interaction-volume simulations to estimate the penetration depth for 15 µm × 15 µm MTJs with 30 nm CFMS as shown in Fig. 1 at decelerated electron-beam voltages (*V*_acc_) of (**a**), 9 and (**b**), 12 kV. The red traces represent the backscattered electron’s (BSE) path. Corresponding scanning electron microscopy (SEM) images are taken by the upper electron detector (UED) with the BSE mode at *V*_acc_ = 10.5 and 11 kV. These two images are subtracted as shown in (**c**), for an MTJ with a large TMR ratio (83%) and (**d**), for an MTJ with a small TMR ratio (18%). Note the line observed in Fig. 2c is a scratch made during the device transfer into SEM.

Electron flights in a multilayered structure were simulated at a series of decelerated electron-beam between 9 and 12 kV using CASINO^6^. The histograms from these simulations are shown in Supplementary Fig. S2. Here, the Co_2_Fe_0.4_Mn_0.6_Si (CFMS)/MgO/CoFe junctions were the layers of interest to correlate any potential defects with the above mentioned TMR reduction. All the layer compositions and their thicknesses were used to simulate the electron flights in the entire multilayers. As shown in Fig. 2a using a 9-kV electron beam, the CASINO simulations confirmed almost 0.002% of the electrons to penetrate into the bottom CFMS layer, while 0.001% and 0.006% were to penetrate the MgO and CoFe layers, respectively. On the other hand, a 12-kV electron beam was calculated to penetrate the CFMS, MgO and CoFe layers by 0.13%, 0.04% and 0.19%, respectively. These simulations gave over 4000% of changes in the penetration probabilities within the CFMS/MgO/CoFe junctions with these decelerated electron-beam voltages (*V*_acc_).

### Imaging with decelerated electron beam

Based on the above simulations, SEM images of the MTJs were taken at the decelerated voltages of 9 and 12 kV as shown in Supplementary Fig. S1. Since our simulations did not take additional parameters, such as film densities, defects and interfacial roughness, further optimisation of *V*_acc_ for imaging was carried out with these voltages. This involved taking a series of images at voltages indicated by the previous simulations. The images that provided the greatest contrast in regions of interest were chosen. A set of two representative images were selected to be taken at *V*_acc_ = 10.5 and 11 kV. The former image taken at *V*_acc_ = 10.5 kV should be predominantly sensitive to any defects above the MgO/CoFe interfaces, while the latter one taken at *V*_acc_ = 11 kV should be primarily sensitive to any defects above the CFMS/MgO interfaces. Hence, by subtracting these two images, one should be able to reconstruct an image representing any defects in the vicinity of the MgO tunnelling barrier.

Figure 2c,d shows the subtracted SEM images taken between *V*_acc_ = 10.5 and 11 keV for the groups (i) and (ii), respectively. It should be noted that both images do not have any defects within the MTJ pillars, which can be observed as grey regions without generating backscattered electrons (BSEs) as imaged previously, suggesting the multilayers are free from interfacial defects. Hence the focus is given to the edges of the pillars. Both images show ~300 nm wide gradient at the edges of the MTJ pillars. This gradient region indicates that the cross-section of the pillars has a shape of trapezium due to the patterning process using Ar-ion milling. In addition, Fig. 2d possesses wider edges at the left edge especially, which suggests the edge may suffer from extra materials redeposited during the milling process. It is important to note that at this stage the change in edge width could just been down to a difference in the incident angle of the Ar-ion beam used to mill the pillars. Chemical analysis done alongside this comparison can help reveal the origin of this oddity.

### Chemical analysis

**Figure 3:**
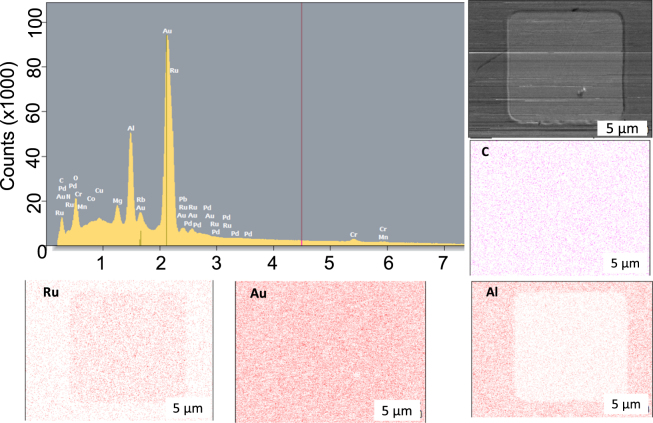
Chemical analysis on an MTJ with a large TMR ratio. Energy dispersive X-ray spectroscopy (EDX) profile for the electron-beam energy between 0.1 and 10 kV for the MTJ. SEM image taken at 10 kV and the corresponding EDX maps are shown for C, Al, Au and Ru.

In order to clarify the origin of the materials attached at the edge, energy dispersive X-ray spectroscopy (EDX) analysis was performed on these two distinctive groups of MTJs. Figure 3, with more examples in Supplementary Fig. S3, shows the EDX signals for the group (i) with large TMR ratios measured at the electron-beam energy between 0.1 and 10 kV and the associated mapping against elements of C, Al, Au and Ru. The strongest signal is from the Au top electrodes, followed by the Al-O insulating layer surrounding the MTJ pillars due to their large volume. Additional minor signals from C, Mg and Ru are appeared in the signal. The EDX mapping confirms that Au is distributed over the surface of the MTJ devices. Al is found to exist only outside the MTJ pillar, while Ru is visible only within the MTJ pillar as expected. It is important to note that C has almost no intensity over the device, indicating no residual resist after the device fabrication and no C-based contamination formed during the analysis.

**Figure 4:**
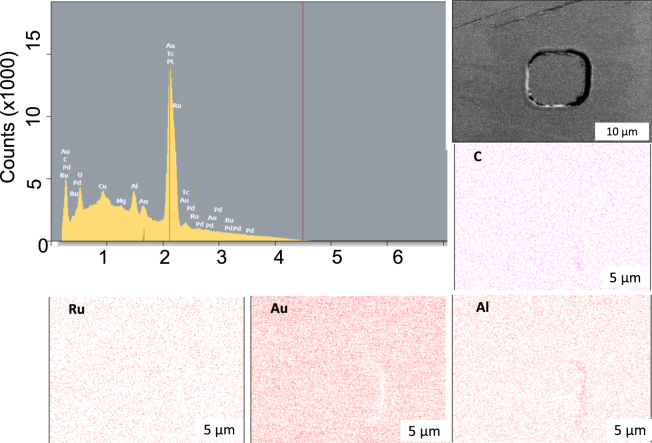
Chemical analysis on an MTJ with a small TMR ratio. Energy dispersive X-ray spectroscopy (EDX) profile for the electron-beam energy between 0.1 and 10 kV for the MTJ. SEM image taken at 10 kV and the corresponding EDX maps are shown for C, Al, Au and Ru.

Figure 4, with further examples in Supplementary Fig. S4, on the other hand, shows the EDX signals for the group (ii) with almost no TMR ratios measured at the electron-beam energy between 0.1 and 10 kV and the associated mapping against elements of C, Al, Au and Ru. The strongest signal is again from the Au top electrodes, followed by the Al-O insulating layers surrounding the MTJ pillars due to their large volume. Additional minor signals from C, Mg and Ru are appeared in the signal as seen in Fig. 3. In the corresponding EDX mapping, Ru is visible only within the MTJ pillar as expected. However, the other elements in EDX mapping shows different results from those in Fig. 3. In Fig. 4, Au distributed over the surface of the MTJ devices except the bottom right edge of the pillar. In this region, Al is found to be concentrated more strongly than that outside the MTJ pillar. Similarly, C is only detected in the same region. This may indicate the formation of an aluminium carbide, such as Al_4_C_3_, at the edge of the MTJ nanopillar. Al_4_C_3_ is known to be formed by the reduction of Al-O at high temperature (~2000 °C). This may suggest residual resist after the Ar-ion milling process may react with Al-O during the Al-O sputtering process. Hence, the resistivity of Al_4_C_3_ has been reported to be 10^2^ Ω cm^7^, which is much smaller than that of MgO, 10^12^ Ω cm. The formation of any aluminium carbides at the edge of a MTJ pillar would cause current shunting through the particles, reducing the corresponding TMR ratio and *RA*.

## Discussion

By comparing the subtracted SEM image and EDX mapping in Figs 2c and 3, the group (i) with large TMR ratios is found to possess smooth interfaces and clean edges. This ensures ideal magnetic transport, achieving the TMR ratio over 80%. On the other hand, Figs 2d and 4 confirm smooth interfaces in the MTJ pillars but with aluminium carbide formation at the edge of the pillars. This introduces a shorting current path, reducing the TMR ratio close to zero. These differences are consistent with the reduction in *RA* by one order of magnitude. The aluminium carbide particles at the MTJ edges are formed by Al-O sputtering onto the residual resist containing carbon. Similar aluminium carbide particles are observed in the other MTJs in the group (ii). The average size of the smallest features are measured to be around 900 nm. By using the resistivity of Al_4_C_3_, *RA* through such a particle is estimated to be 9 × 10^5^ Ω µm^2^. In the case of the 5 nm CFMS devices we managed to improve the yield from 21% to 36% by reducing the beam current during the Al-O deposition, with details discussed in the supplementary section and results shown in ST3. The EDX mapping with decelerated electron-beam imaging therefore allow further process optimisation of junction fabrication to sustain the advancement in their density and functionality at low cost.

## Methods

### Device fabrication

Multilayers, consisting of Cr (80)/Pd (5)/Co_2_Fe_0.4_Mn_0.6_Si (5 or 30)/MgO (2)/Co_0.5_Fe_0.5_ (5)/IrMn_3_ (10)/Ru (7) (thickness in nm), were grown on MgO(001) substrates at room temperature using an ultrahigh vacuum (UHV) sputtering system (ULVAC, MPS series)^8^. After the deposition of the CFMS layer, the entire stack was annealed at 400 °C for 20 minutes in the UHV system to crystallise the CFMS Heusler alloy layer into the perfectly-ordered *L*2_1_ phase.

The structure listed above, and shown in Fig. 1, was chosen to maximise TMR effects and also allow for simple transport measurements. The bottom Cr and Pd layers act simultaneously as seed layers, to promote the *L*2_1_ phase, and electrodes for measurement. The CFMS is the free layer, with the CoFe acting as it’s reference layer and the MgO as the tunnel barrier. The IrMn_3_ provides exchange bias, fixing the reference layer. Finally the Ru, Cr and Au act as both capping layers and lead electrodes for the system.

The multilayered stacks were then patterned into MTJs with their area between 10 µm × 10 µm and 50 µm × 50 µm using photolithography and Ar-ion milling. Here, photoresist of AZ5214E (Micro Chemicals) with typical thickness of 1.4 µm was spin-coated on the multilayers to create positive patterns of bottom electrodes. The patterns were then milled by Ar^+^ ions (Hakuto, 10IBE), followed by the removal of the remaining resist using N-methylpyrrolidone. On these bottom electrodes, MTJ pillars were patterned using the same milling process with end-point detection just below the Pd/CFMS interfaces. The milled regions were filled with Al-O by UHV sputtering, followed by the lift-off with N-methylpyrrolidone. The top electrodes of Cr (5)/Au (80) (thickness in nm) were finally deposited by UHV sputtering through similarly made patterns by photolithography, followed by the similar lift-off process.

### Magnetotransport measurement

The transport properties of the MTJs were assessed by four-terminal magnetoresistance measurements. Here, an electrical current of about 100 µA and an external magnetic field of up to ±50 mT along the <110> direction of the CFMS layer were applied during the measurements at room temperature. The details of the magnetotransport properties have been published elsewhere^9^.

### SEM observation

In the JSM-7800F Prime SEM^10^, an upper electron detector (UED) is located above the lens and two additional detectors, a retractable backscattered electron detector (BED) and a lower electron detector (LED), are installed just above the sample space. The UED becomes sensitive to reflected electrons from the specimen with energies above 10 eV when a bias voltage (*V*) is applied to the energy filter beneath, while the USD senses those below 1 eV. These detection modes with energy deceleration in the range between +500 eV and −2 keV can be achieved coincidently. In addition, the acceleration voltage for the electron-beam can be controlled between 10 and 30 kV^10^. Based on such controllability, almost 0.7-nm resolution is guaranteed at the effective electron-beam landing voltages of both 1 and 5 kV at the specimen by the manufacturer.

### Data availability

The data that support the findings of this study are available from the corresponding author upon request.

## Electronic supplementary material

Supplementary Information
