# Supplementary material for: Chemical and structural analysis on magnetic tunnel junctions using a decelerated scanning electron beam
Source: Sci Rep. 2018 May 15;8:7585. doi: 10.1038/s41598-018-25638-8 (PMC5954102; doi:10.1038/s41598-018-25638-8)
Supplement: Supplementary file 1 — Supplementary Information [file 41598_2018_25638_MOESM1_ESM.pdf]

Chemical and structural analysis on magnetic tunnel junctions using a decelerated scanning electron beam

Edward Jackson,[1] Mingling Sun,[2] Takahide Kubota,[2,3] Koki Takanashi [2,3] and Atsufumi Hirohata [1,*]

[1] *Department of Electronic Engineering, University of York, Heslington, York YO10 5DD, United Kingdom*
[2] *Institute for Materials Research, Tohoku University, 2-1-1 Katahira, Sendai 980-8577, Japan*
[3] *Center for Spintronics Research Network, Tohoku University, 2-1-1 Katahira, Sendai 980-8577, Japan*
* e-mail: atsufumi.hirohata@york.ac.uk

Supplementary information

Figure S1 shows raw SEM images taken at the electron-beam acceleration voltages between 9 and 12 kV. As seen in Fig. S1a, there are two circular defects observed in the middle of the MTJ nanopillar, which become significant in Fig. S1b but diminish in Figs. S1c and d. By making electron flight simulations on these acceleration voltages as shown in Fig. S2, the defects are formed in the layers between the top Cr and CoFe, which may not change the magnetic transport properties across CoFe/MgO/$Co_2Fe_{0.4}Mn_{0.6}Si$ (CFMS). Additional vertical stripe features are seen in Fig. S1. Since these stripe features are observed in all four SEM images, they may be induced by the initial interface between the MgO substrate and the Cr seed layer. Hence, no major defects are formed across the CFMS/MgO/CoFe interfaces. These images are subtracted for comparison as discussed in the main text, to ascertain which voltages should be utilised for more intensive analysis of the interface of interest, the MgO barrier.

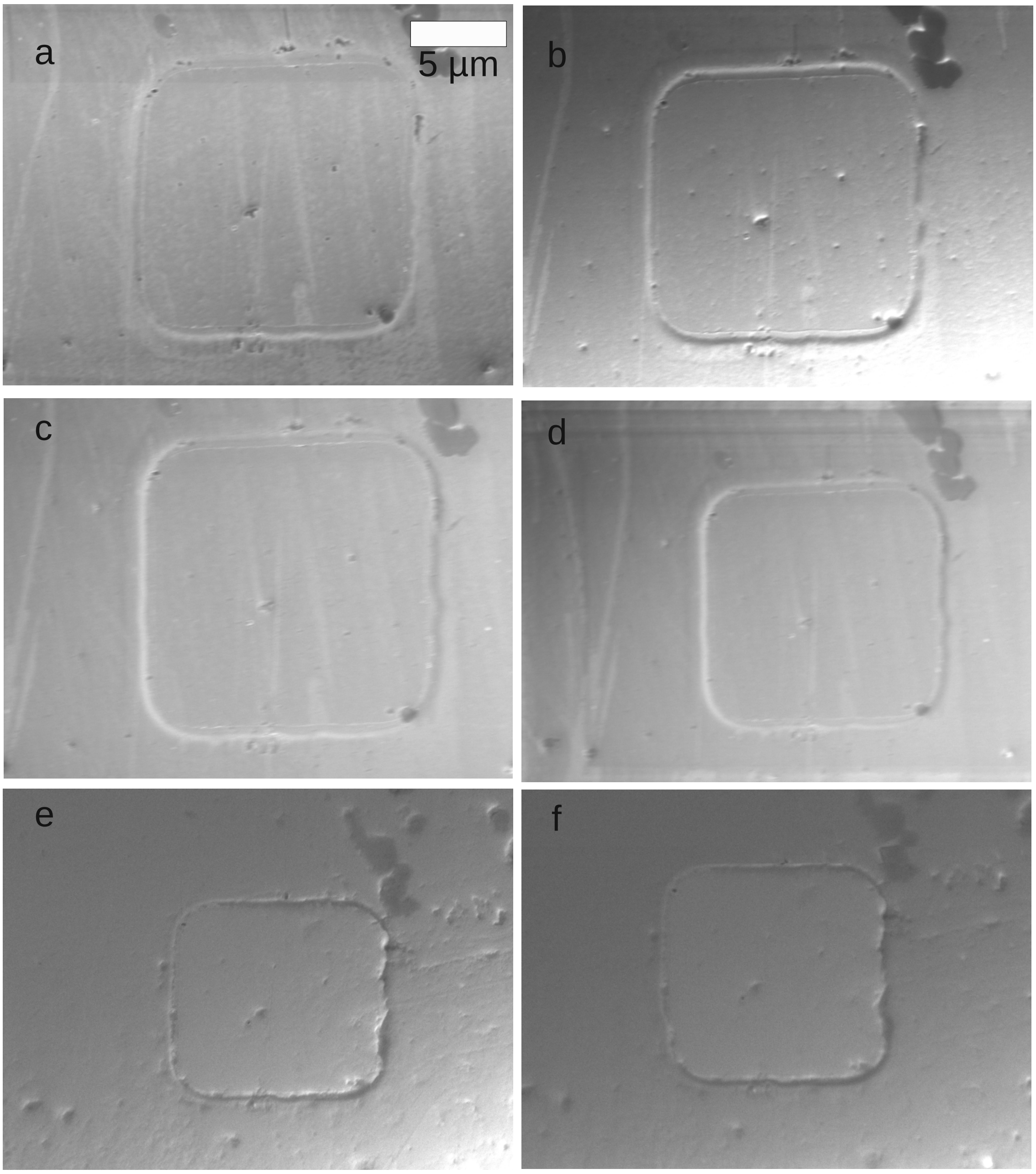

Figure S1: Decelerated beam images. A series of images taken of one of the magnetic tunnel junctions, consisting of MgO(001)//Cr (80)/Pd (5)/$Co_2Fe_{0.4}Mn_{0.6}Si$ (5)/MgO (2)/$Co_{0.5}Fe_{0.5}$ (5)/$IrMn_3$ (10)/Ru (7)/Cr (5)/Au (80) (thickness in nm). This device is 15 µm × 15 µm imaged at the acceleration beam voltages of **a,** 9, **b,** 9.5, **c,** 10, **d,** 10.5, **e,** 11 and **f,** 12 kV.

As can be seen in these histograms the number of electrons interacting with the areas of interest (MgO/CFMS/CoFe) greatly increase with increasing acceleration voltages. In this system the 80nm Au cap acts as a very large barrier for electrons. As the voltage increases larger numbers can overcome this barrier drastically increasing the number of BSE interactions.

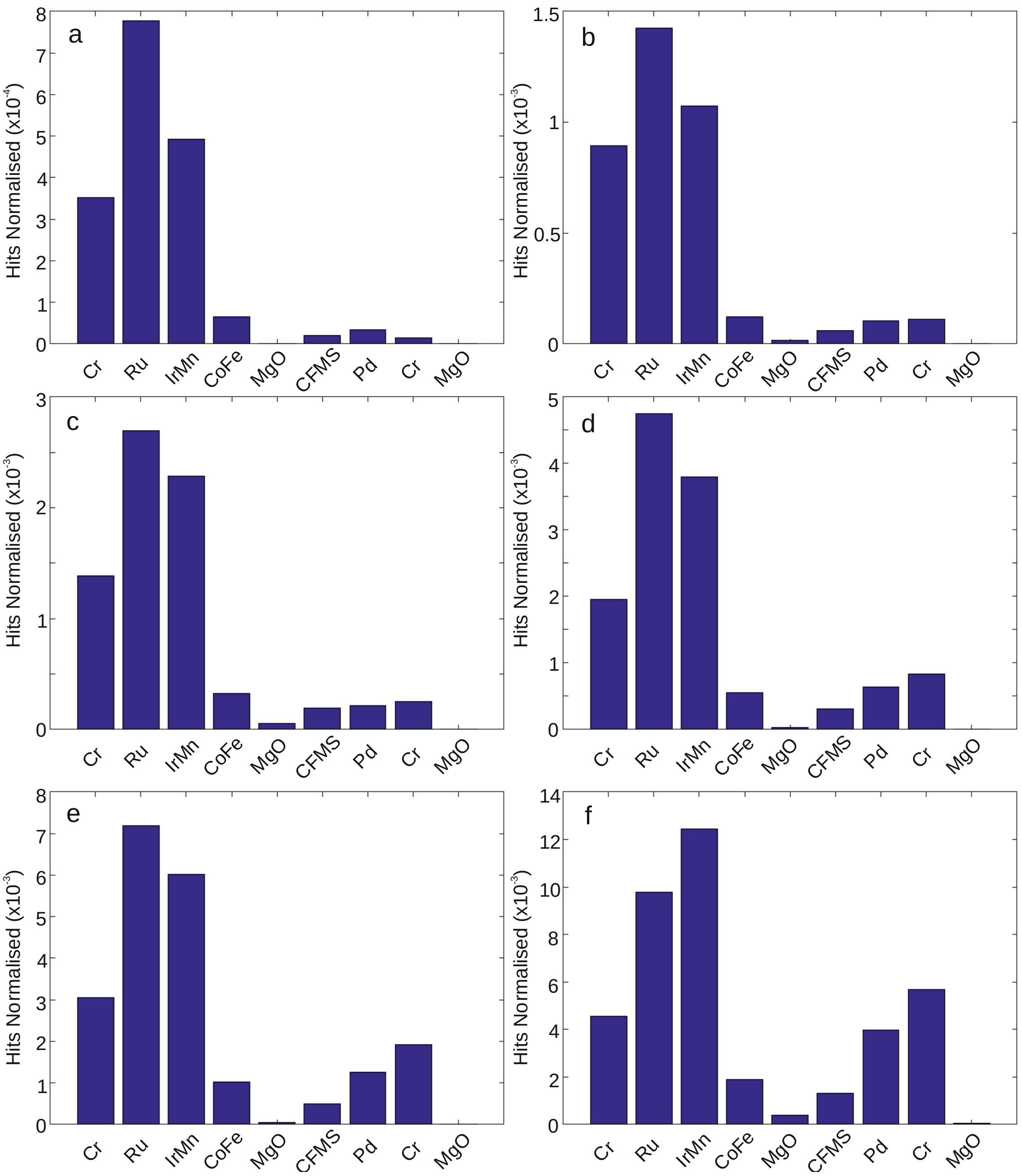

Figure S2: Decelerated beam simulations. A series of histograms of backscattered electrons to be generated from a magnetic tunnel junctions, consisting of MgO(001)//Cr (80)/Pd (5)/$Co_2Fe_{0.4}Mn_{0.6}Si$ (5)/MgO (2)/$Co_{0.5}Fe_{0.5}$ (5)/$IrMn_3$ (10)/Ru (7)/Cr (5)/Au (80) (thickness in nm) at the acceleration beam voltages of **a,** 9, **b,** 9.5, **c,** 10, **d,** 10.5, **e,** 11 and **f,** 12 kV. The remainder of the BSEs are found in the gold capping layer.

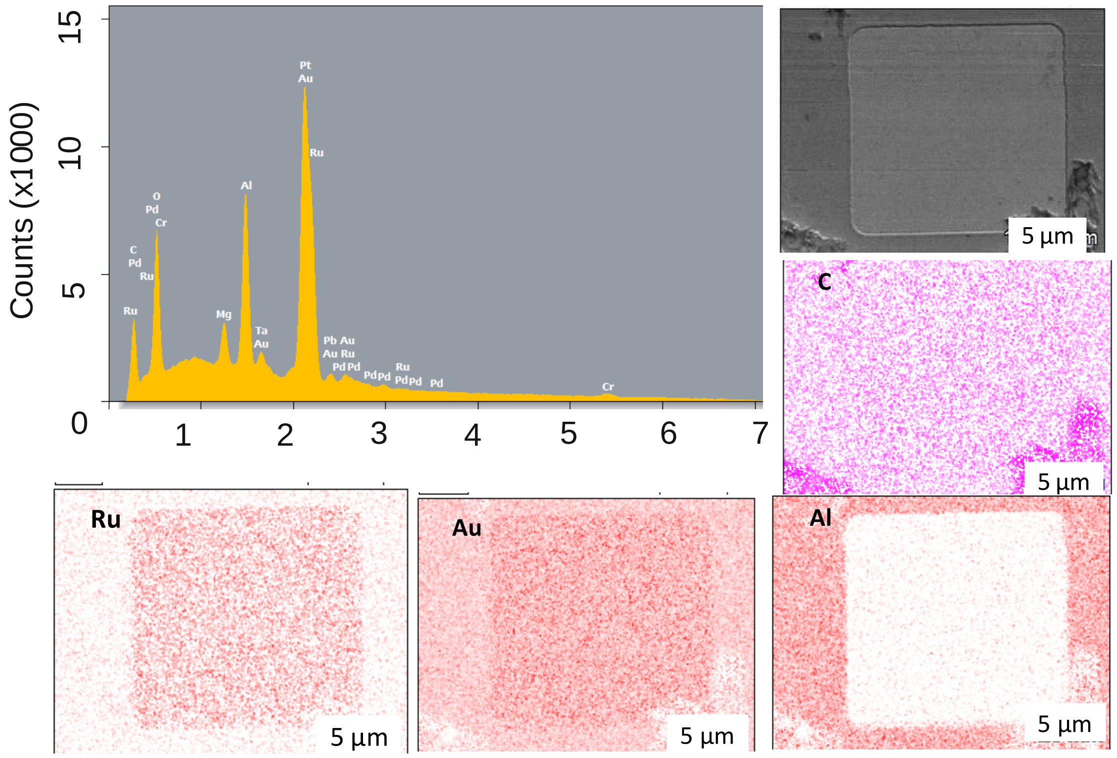

Figure S3: High TMR Pillar EDX. Further EDX profiles for a high TMR pillar. SEM image taken at 10 kV and the corresponding EDX maps are shown for C, Al, Au and Ru. In this case strong carbon surface contaminants can be seen, but despite this the same structures appear. Ru is confined to the pillar and Al is kept outside of it as expected.

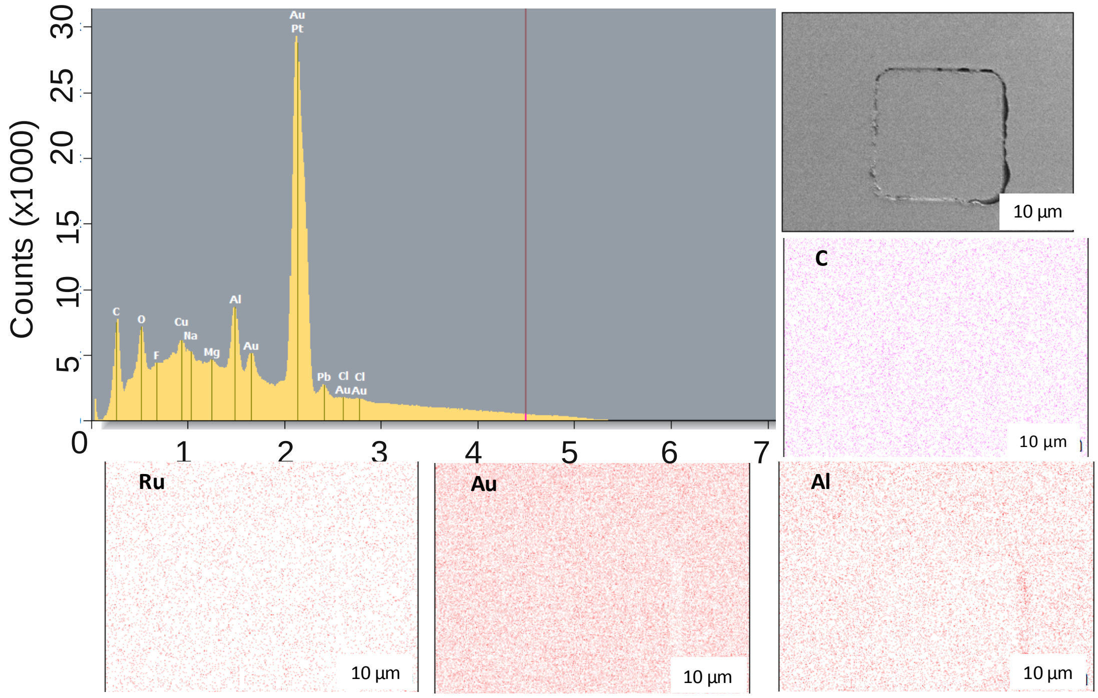

Figure S4: low TMR Pillar EDX. Further EDX profiles for a low TMR pillar. SEM image taken at 10 kV and the corresponding EDX maps are shown for C, Al, Au and Ru. In this case the Al's most well defined feature is along the edge of the pillar, which can be correlated to the SEM image and the lower concentration of Au.

To test the hypothesis that aluminium carbide formation during the deposition of aluminium oxide is reducing the yield of the devices studied a second set of similar samples were produced. In this set the aluminium oxide was deposited at 60% of the original current. In the 1st set of samples, tabulated in ST1, the yield was 69%. This was defined by the number of devices with a MR ≥ 80%, dived by the total number of devices produced (42). For the 2nd set, with a lower beam current, this yield dropped to 12%.

Size (μm^2)<br>MR(%)<br>RA (Ωμm^2)	50 x 50<br>80.20<br>2.56 x10^4	50 x 50<br>95.2<br>3.56 x10^4	50 x 50<br>73.2<br>2.02 x10^4	50 x 50<br>94.7<br>4.38 x10^4	50 x 50<br>87.1<br>3.54 x10^4	50 x 50<br>0<br>0
Size (μm^2)<br>MR(%)<br>RA (Ωμm^2)	40 x 40<br>86.2<br>3.13 x10^4	40 x 40<br>94.8<br>3.68 x10^4	40 x 40<br>92.9<br>3.55 x10^4	40 x 40<br>53.2<br>1.25 x10^4	40 x 40<br>83.4<br>3.06 x10^4	40 x 40<br>93.8<br>3.87 x10^4
Size (μm^2)<br>MR(%)<br>RA (Ωμm^2)	30 x 30<br>94.1<br>3.72 x10^4	30 x 30<br>83<br>2.80 x10^4	30 x 30<br>7<br>2.09 x10^2	30 x 30<br>18.4<br>1.53 x10^3	30 x 30<br>96.4<br>4.19 x10^4	30 x 30<br>97.7<br>4.16 x10^4
Size (μm^2)<br>MR(%)<br>RA (Ωμm^2)	25 x 25<br>28.59<br>8.79 x10^3	25 x 25<br>87.4<br>3.18 x10^4	25 x 25<br>95<br>3.69 x10^4	25 x 25<br>44.6<br>8.38 x10^3	25 x25<br>33.4<br>4.96 x10^3	25 x 25<br>97.7<br>4.06 x10^4
Size (μm^2)<br>MR(%)<br>RA (Ωμm^2)	20 x 20<br>95.4<br>3.67 x10^4	20 x 20<br>96.83<br>3.72 x10^4	20 x 20<br>95.9<br>3.70 x10^4	20 x 20<br>73.5<br>2.19 x10^4	20 x 20<br>96.7<br>3.97 x10^4	20 x 20<br>95.3<br>3.82 x10^4
Size (μm^2)<br>MR(%)<br>RA (Ωμm^2)	15 x 15<br>75.4<br>2.34 x10^4	15 x 15<br>96.8<br>3.52 x10^4	15 x 15<br>93.7<br>3.45 x10^4	15 x 15<br>91.3<br>3.43 x10^4	15 x 15<br>96.1<br>3.83 x10^4	15 x 15<br>96.5<br>3.78 x10^4
Size (μm^2)<br>MR(%)<br>RA (Ωμm^2)	10 x 10<br>95.1<br>3.33 x10^4	10 x 10<br>94.4<br>3.19 x10^4	10 x 10<br>0<br>0	10 x 10<br>23.9<br>2.24 x10^3	10 x 10<br>44.4<br>5.97 x10^3	10 x 10<br>95.8<br>3.40 x10^4

Table ST1: Transport measurements for the devices with MgO(001)//Cr (80)/Pd (5)/$Co_2Fe_{0.4}Mn_{0.6}Si$ (30)/MgO (2)/$Co_{0.5}Fe_{0.5}$ (5)/$IrMn_3$ (10)/Ru (7)/Cr (5)/Au (80) (thickness in nm). If the devices are grouped by their MR, either high or low, the groups will contain devices from the full range of sizes.

A						
Size (μm^2)<br>MR(%)<br>RA (Ωμm^2)	50 x 50<br>34.4<br>3.85 x10^4	50 x 50<br>25.9<br>2.98 x10^4	50 x 50<br>46.5<br>5.05 x10^4	50 x 50<br>107.0<br>7.83 x10^4	50 x 50<br>38.2<br>4.21 x10^4	50 x 50<br>32.7<br>3.85 x10^4
Size (μm^2)<br>MR(%)<br>RA (Ωμm^2)	40 x 40<br>25.7<br>3.14 x10^4	40 x 40<br>67.9<br>6.02 x10^4	40 x 40<br>23.7<br>3.06 x10^4	40 x 40<br>54.2<br>5.17 x10^4	40 x 40<br>21.3<br>2.53 x10^4	40 x 40<br>42.4<br>4.42 x10^4

Size (μm^2)<br>MR(%)<br>RA (Ωμm^2)	30 x 30<br>8.6<br>1.06 x10^4	30 x 30<br>51<br>4.97 x10^4	30 x 30<br>32<br>9.86 x10^4	30 x 30<br>51.2<br>4.91 x10^4	30 x 30<br>38.4<br>4.12 x10^4	30 x 30<br>47.2<br>4.70 x10^4
Size (μm^2)<br>MR(%)<br>RA (Ωμm^2)	25 x 25<br>24.9<br>2.96 x10^4	25 x 25<br>9.6<br>1.27 x10^4	25 x 25<br>30.5<br>3.59 x10^4	25 x 25<br>8.0<br>8.94 x10^3	25 x25<br>7.2<br>8.63 x10^3	25 x 25<br>84.9<br>6.33 x10^4
Size (μm^2)<br>MR(%)<br>RA (Ωμm^2)	20 x 20<br>46.2<br>4.66 x10^4	20 x 20<br>63.6<br>5.42 x10^4	20 x 20<br>97.1<br>7.53 x10^4	20 x 20<br>101.4<br>6.62 x10^4	20 x 20<br>7.8<br>5.15 x10^3	20 x 20<br>83.2<br>6.24 x10^4
Size (μm^2)<br>MR(%)<br>RA (Ωμm^2)	15 x 15<br>19.3<br>2.30 x10^4	15 x 15<br>95.0<br>6.99 x10^4	15 x 15<br>6<br>7.76 x10^3	15 x 15<br>97.0<br>6.14 x10^4	15 x 15<br>46.8<br>4.22 x10^4	15 x 15<br>22.6<br>2.32 x10^4
Size (μm^2)<br>MR(%)<br>RA (Ωμm^2)	10 x 10<br>9.7<br>1.11 x10^4	10 x 10<br>11.5<br>1.34 x10^4	10 x 10<br>6<br>6.56 x10^3	10 x 10<br>72.6<br>4.99 x10^4	10 x 10<br>87.4<br>5.49 x10^4	10 x 10<br>85.2<br>5.50 x10^4
B						
Size (μm^2)<br>MR(%)<br>RA (Ωμm^2)	50 x 50<br>95.7<br>5.20 x10^4	50 x 50<br>8.7<br>5.50 x10^3	50 x 50<br>60.4<br>3.83 x10^4	50 x 50<br>8.20<br>6.50 x10^3	50 x 50<br>15.5<br>1.20 x10^4	50 x 50<br>95.5<br>4.95 x10^4
Size (μm^2)<br>MR(%)<br>RA (Ωμm^2)	40 x 40<br>93.3<br>4.90 x10^4	40 x 40<br>6.5<br>5.76 x10^3	40 x 40<br>95.3<br>4.99 x10^4	40 x 40<br>15.9<br>1.22 x10^4	40 x 40<br>13.7<br>1.14 x10^4	40 x 40<br>3.7<br>3.04 x10^3
Size (μm^2)<br>MR(%)<br>RA (Ωμm^2)	30 x 30<br>92.3<br>4.59 x10^4	30 x 30<br>92.3<br>4.73 x10^4	30 x 30<br>16.9<br>1.39 x10^4	30 x 30<br>90.3<br>4.67 x10^4	30 x 30<br>90.3<br>4.64 x10^4	30 x 30<br>24.2<br>1.80 x10^4
Size (μm^2)<br>MR(%)<br>RA (Ωμm^2)	25 x 25<br>89.0<br>4.66 x10^4	25 x 25<br>31.5<br>2.24 x10^4	25 x 25<br>91<br>4.63 x10^4	25 x 25<br>87.8<br>4.51 x10^4	25 x25<br>10.3<br>7.88 x10^3	25 x 25<br>90.0<br>4.36 x10^4
Size (μm^2)<br>MR(%)<br>RA (Ωμm^2)	20 x 20<br>26.1<br>1.98 x10^4	20 x 20<br>26.2<br>1.88 x10^4	20 x 20<br>88.2<br>4.46 x10^4	20 x 20<br>85.1<br>4.16 x10^4	20 x 20<br>12.3<br>8.56 x10^3	20 x 20<br>12.4<br>8.92 x10^3
Size (μm^2)<br>MR(%)<br>RA (Ωμm^2)	15 x 15<br>14.2<br>1.13 x10^4	15 x 15<br>12.9<br>6.93 x10^3	15 x 15<br>79.0<br>4.14 x10^4	15 x 15<br>84.3<br>2.56 x10^4	15 x 15<br>21.6<br>1.40 x10^4	15 x 15<br>52.4<br>2.16 x10^4
Size (μm^2)<br>MR(%)<br>RA (Ωμm^2)	10 x 10<br>0<br>1.85 x10^3	10 x 10<br>66.4<br>3.94 x10^4	10 x 10<br>79.3<br>2.00 x10^4	10 x 10<br>61.2<br>3.95 x10^4	10 x 10<br>62.0<br>3.90 x10^4	10 x 10<br>62.3<br>3.82 x10^4

Table ST2: Transport measurements for the devices with MgO(001)//Cr (20)/Ru(50)/$Co_2Fe_{0.4}Mn_{0.6}Si$ (5)/MgO (2)/$Co_{0.5}Fe_{0.5}$ (5)/$IrMn_3$ (10)/Ru (7)/Cr (5)/Au (80)

(thickness in nm). (A) shows the devices which had aluminium oxide deposited at 50 mA. (B) shows the devices which had aluminium oxide deposited at 30 mA.

This drop can be explained by the increase in difficulty of lift-off due to the hardening of the photoresist. This hardening occurred because the cooling of the stage was insufficient for such a long deposition time. With this in mind the CFMS = 5 nm group of samples, which are tabulated in ST2, were investigated in a similar manner. As they were shorter stacks, even at the lower deposition current they would not take long enough for the cooling to become inadequate. In this case the 1st set of samples had a yield of 21%, whilst the 2nd set had a yield of 36%. This increase demonstrates that one of the issues this structure faced was the formation of aluminium carbide, which allowed us to remove one of the problems in device fabrication. A summary of this yield test is shown in Table ST3.

Device Group	30 - 1st	30 - 2nd	5 – 1st	5 – 2nd
Beam Current (mA)	50	30	50	30
Deposition time (mins)	47	87	36	65
Yield (%)	69	12	21	36

Table ST3: Yield of devices with MR ≥ 80%. The groups are listed based on their CFMS thickness, either 5 or 30. All were deposited at 1000 V.
